# Supplementary material for: Characterization of Serum and Mucosal SARS-CoV-2-Antibodies in HIV-1-Infected Subjects after BNT162b2 mRNA Vaccination or SARS-CoV-2 Infection
Source: Viruses. 2022 Mar 21;14(3):651. doi: 10.3390/v14030651 (PMC8952283; doi:10.3390/v14030651)
Supplement: Supplementary file 1 [file viruses-14-00651-s001.zip › Suppl. Table S1.pdf]

Supplementary Table S1: Characteristics of study subjects

ID	Sex	Age	COVID	Severity	Vaccination	Days between doses	Days post Boost/ Infection	HIV-Infection	Years infected	Years under therapy	Viral load	Therapy	CD4+ T-cells	CD8+ T-cells	Comorbidities
224	M	44	N		N			Y	8	8	<20	FTC/TAF/RPV	429	568	HyT
539	F	36	N		N			Y	5	5	30	DRV, RTV, DTG, FTC	468	684	N
93	M	35	N		N			Y	5	5	<20	FTC/TAF/BIC	921	1020	PCBCL 2015, ASCT 2016
540	M	54	N		N			Y	9	9	<20	FTC/TAF/BIC	642	489	N
356	M	34	N		N			Y	1	1	<20	FTC/TAF/BIC	499	1189	N
70	M	56	N		N			Y	29	24	20	FTC/TAF/COB /EVG	728	664	N
254	F	44	N		Y	21	34	N				None	N/A	N/A	N[1]
264	M	35	N		Y	21	9	N				None	N/A	N/A	N
269	M	60	N		Y	21	14	N				None	N/A	N/A	N
265	F	27	N		Y	21	14	N				None	N/A	N/A	N
268	F	32	N		Y	21	14	N				None	N/A	N/A	N
266	F	43	N		Y	21	14	N				None	N/A	N/A	N
267	M	25	N		Y	21	14	N				None	N/A	N/A	N
271	F	56	N		Y	26	23	N				None	N/A	N/A	N
270	M	61	N		Y	21	36	N				None	N/A	N/A	N
6	F	25	N		Y	21	17	N				None	N/A	N/A	N

ID	Sex	Age	COVID	Severity	Vaccination	Days between doses	Days post Boost/ Infection	HIV-Infection	Years infected	Years under therapy	Viral load	Therapy	CD4+ T-cells	CD8+ T-cells	Comorbidities
272	F	23	N		Y	21	20	N				None	N/A	N/A	N
8	F	44	N		Y	21	17	N				None	N/A	N/A	N
5	F	60	N		Y	21	20	N				None	N/A	N/A	N
15	F	23	N		Y	21	78	N				None	N/A	N/A	N
2	M	60	N		Y	21	119	N				None	N/A	N/A	N
526	M	80	N		Y	21	8	Y	22	22	<20	FTC/TAF/BIC	795	1481	AF, CHD
56	F	58	N		Y	21	20	Y	25	19	<20	FTC/TAF/COB /EVG	578	561	N
74	M	32	N		Y	21	60	Y	7	7	<20	FTC/TAF/BIC	928	1228	N
232	F	63	N		Y	21	16	Y	32	15	<20	3TC/ABC/DTG	694	642	CT, HPT
383	M	36	N		Y	22	98	Y	6	6	20	FTC/TAF/COB /EVG	368	1067	N
275	M	52	N		Y	35	16	Y	19	5	20	FTC/TAF/BIC	904	1440	N
420	F	63	N		Y	21	111	Y	35	19	<20	FTC/TAF/COB /EVG	426	586	OUD
45	M	68	N		Y	42	8	Y	17	17	<20	FTC/TAF/COB /EVG	473	311	CHD
496	M	52	N		Y	43	19	Y	11	11	20	FTC/TAF/COB /DRV	1443	1948	BL
240	M	54	N		Y	21	38	Y	16	16	<20	ABC/3TC, EFV	905	861	N
503	M	58	N		Y	43	28	Y	½	½	30	DTG, FTC/TDF	300	1321	N
98	M	55	N		Y	42	43	Y	10	9	<20	ABC/3TC/DTG	555	685	N

ID	Sex	Age	COVID	Severity	Vaccination	Days between doses	Days post Boost/ Infection	HIV-Infection	Years infected	Years under therapy	Viral load	Therapy	CD4+ T-cells	CD8+ T-cells	Comorbidities
390	M	68	N		Y	21	75	Y	5	5	20	FTC/TAF/COB /DRV, DTG	343	566	DM2
289	M	36	N		Y	42	22	Y	4	4	<20	FTC/TAF/BIC	596	522	N
436	M	65	N		Y	42	35	Y	4	4	20	FTC/TAF/BIC	335	658	COPD, PAOD
396	M	60	N		Y	42	76	Y	7	7	20	FTC/TAF/BIC	234	1042	DM2, SA
218	M	61	N		Y	42	62	Y	8	8	<20	DRV, RTV, ABC/3TC/DTG	269	517	N
297	M	60	N		Y	42	61	Y	4	4	20	ABC/3TC/DTG	266	108	N
441	M	44	N		Y	40	155	Y	2	2	20	FTC/TAF/BIC	31	448	N
256	M	29	Y	3	N		100	N				None	N/A	N/A	N
263	M	35	Y	2	N		72	N				None	N/A	N/A	N
565	M	32	Y	2	N		107	N				None	N/A	N/A	N
566	M	34	Y	2	N		179	N				None	N/A	N/A	N
567	F	36	Y	3	N		154	N				None	N/A	N/A	N
307	M	25	Y	3	Y		158	N				None	N/A	N/A	N
568	M	49	Y	3	N		77	N				None	N/A	N/A	N
569	F	50	Y	3	N		77	N				None	N/A	N/A	N
113	M	63	Y	1	Y	2d post 1st dose	199	Y	23	23	<20	FTC/TAF/BIC	563	1028	N
334	M	31	Y	3	N		103	Y	2	2	20	DTG, FTC/TDF	350	665	N

ID	Sex	Age	COVID	Severity	Vaccination	Days between doses	Days post Boost/ Infection	HIV-Infection	Years infected	Years under therapy	Viral load	Therapy	CD4+ T-cells	CD8+ T-cells	Comorbidities
532	M	28	Y	3	N		51	Y	8	8	<20	FTC/TDF/RPV	814	668	AA
501	M	41	Y	3	N		73	Y	23	16	<20	FTC/TAF/BIC	44	84	LCpH
230	F	31	Y	3	N		136	Y	7	7	<20	FTC/TDF/RPV	688	946	N
120	M	46	Y	2	N		153	Y	5	5	<20	FTC/TAF/BIC	538	723	N
178	M	45	Y	3	N		57	Y	4	4	20	3TC/ABC/DTG	470	632	N
492	F	41	Y	1	N		38	Y	12	12	<20	FTC/TAF/COB /DRV, DTG	505	379	N
491	M	50	Y	4	N		93	Y	23	23	<20	ETR, RAL, DRV, RTV	274	440	HbH
422	F	66	Y	2	N		28	Y	25	11	<20	ABC/3TC, RAL	625	447	CC
552	M	52	Y	2	N		175	Y	16	16	<20	FTC/TAF/BIC	341	458	N
260	M	49	Y	2	N		164	Y	2	2	<20	FTC/TAF/BIC	405	778	N
564	F	25	Y	2	N		59	Y	1	1	<20	FTC/TAF, DTG	210	360	DM1
1	F	61	N		Y	21	14	N				None	N/A	N/A	N
247	M	38	N		Y	21	15	N				None	N/A	N/A	N
248	M	53	N		Y	21	15	N				None	N/A	N/A	N
251	M	61	N		Y	21	16	N				None	N/A	N/A	N
252	F	54	N		Y	21	9	N				None	N/A	N/A	N
9	M	23	N		Y	21	21	N				None	N/A	N/A	N

ID	Sex	Age	COVID	Severity	Vaccination	Days between doses	Days post Boost/ Infection	HIV-Infection	Years infected	Years under therapy	Viral load	Therapy	CD4+ T-cells	CD8+ T-cells	Comorbidities
514	F	48	N		Y	21	27	N				None	N/A	N/A	N
516	F	50	N		Y	22	68	N				None	N/A	N/A	N
517	F	45	N		Y	21	68	N				None	N/A	N/A	N
528	M	30	N		Y	23	20	N				None	N/A	N/A	N
475	F	37	N		Y	23	23	N				None	N/A	N/A	N
580	M	53	N		Y	28	37	N				None	N/A	N/A	N
4083	F	23	N		Y	32	20	N				None	N/A	N/A	N
4439	F	30	N		Y	42	23	N				None	N/A	N/A	N
3012	F	34	N		Y	42	46	N				None	N/A	N/A	N
3865	F	68	N		Y	42	22	N				None	N/A	N/A	N
3815	F	54	N		Y	49	76	N				None	N/A	N/A	N
4402	F	47	N		Y	36	114	N				None	N/A	N/A	N
2918	M	27	N		Y	N/A	59	N				None	N/A	N/A	N
4628	M	34	N		Y	42	62	N				None	N/A	N/A	N
908	M	35	N		Y	42	87	N				None	N/A	N/A	N
3827	M	40	N		Y	27	49	N				None	N/A	N/A	N
89	M	44	N		Y	42	155	N				None	N/A	N/A	N

ID	Sex	Age	COVID	Severity	Vaccination	Days between doses	Days post Boost/ Infection	HIV-Infection	Years infected	Years under therapy	Viral load	Therapy	CD4+ T-cells	CD8+ T-cells	Comorbidities
770	M	45	N		Y	22	25	N				None	N/A	N/A	N
773	M	53	N		Y	23	29	N				None	N/A	N/A	N
781	M	48	N		Y	22	43	N				None	N/A	N/A	N
9240	M	41	N		Y	42	77	N				None	N/A	N/A	N
4432	M	35	N		Y	30	12	N				None	N/A	N/A	N
1452	M	38	N		Y	42	15	N				None	N/A	N/A	N
1674	F	53	N		Y	39	84	N				None	N/A	N/A	N
2998	F	29	N		Y	42	49	N				None	N/A	N/A	N
3959	M	30	N		Y	36	92	N				None	N/A	N/A	N
2799	M	34	N		Y	25	19	N				None	N/A	N/A	N
7673	M	26	N		Y	42	61	N				None	N/A	N/A	N
2960	M	42	N		Y	21	139	N				None	N/A	N/A	N
1694	M	50	N		Y	43	81	N				None	N/A	N/A	N
3830	M	52	N		Y	49	76	N				None	N/A	N/A	N
1657	M	53	N		Y	42	99	N				None	N/A	N/A	N
2974	M	24	N		Y	42	58	N				None	N/A	N/A	N
1827	M	27	N		Y	24	72	N				None	N/A	N/A	N

ID	Sex	Age	COVID	Severity	Vaccination	Days between doses	Days post Boost/ Infection	HIV-Infection	Years infected	Years under therapy	Viral load	Therapy	CD4+ T-cells	CD8+ T-cells	Comorbidities
7864	F	27	N		Y	42	48	N				None	N/A	N/A	N
530	M	32	N		Y	23	16	N				None	N/A	N/A	N
273	F	50	N		Y	22	25	N				None	N/A	N/A	N
487	M	23	N		Y	27	14	N				None	N/A	N/A	N
23	F	53	N		Y	21	15	N				None	N/A	N/A	N
538	M	41	N		Y	23	20	Y	7	7	<20	DOR, DTG, ABC	831	773	CKD, DM1, HyT
469	M	72	N		Y	40	10	Y	24	24	20	AZV, ABC/3TC	910	550	DM2
305	M	59	N		Y	21	41	Y	10	10	<20	FTC/TAF/BIC	257	588	COPD, HyT, PAOD, PF
562	M	37	N		Y	21	50	Y	12	9	20	ABC/3TC/DTG	1488	1410	D
563	M	47	N		Y	21	50	Y	9	8	20	ABC/3TC/DTG	1240	389	N
141	F	55	N		Y	40	42	Y	36	30	<20	DRV,RTV, DTG/RPV	558	279	ACL, LF
143	F	24	N		Y	42	7	Y	2	2	<20	FTC/TDF,RAL	868	620	N
183	M	62	N		Y	42	14	Y	19	19	<20	ABC/3TC/DTG	671	373	DM2
462	M	30	N		Y	26	55	Y	3	3	<20	FTC/TAF/COB /DRV	329	726	N
340	M	51	N		Y	42	13	Y	12	12	<20	3TC/DTG	970	891	N
571	M	46	N		Y	42	39	Y	5	5	20	FTC/TAF/BIC	512	351	N
329	M	36	N		Y	42	36	Y	11	5	20	ABC/3TC/DTG	792	815	N

ID	Sex	Age	COVID	Severity	Vaccination	Days between doses	Days post Boost/ Infection	HIV-Infection	Years infected	Years under therapy	Viral load	Therapy	CD4+ T-cells	CD8+ T-cells	Comorbidities
65	M	55	N		Y	42	21	Y	7	7	20	FTC/TDF, EFV	1058	595	N
119	M	53	N		Y	49	28	Y	18	14	<20	FTC/TAF/BIC	1014	1201	N
154	M	53	N		Y	42	36	Y	13	13	40	FTC/TAF/BIC	351	628	D
336	M	35	N		Y	42	30	Y	8	8	20	FTC/TAF/COB /DRV, DTG	946	1270	N
509	M	53	N		Y	41	23	Y	5	5	20	FTC/TAF/BIC	471	656	N
323	F	45	N		Y	21	11	Y	19	19	<20	FTC/TDF, DTG	342	270	N
485	M	51	N		Y	42	30	Y	27	27	<20	ATV,COB,ETR ,RAL	740	648	N
158	F	54	N		Y	42	48	Y	27	27	<20	3TC/DTG,DRV ,RTV	877	292	N
239	F	51	N		Y	41	62	Y	7	7	<20	FTC/TAF/COB /DRV	286	504	N
46	M	56	N		Y	42	77	Y	4	4	<20	ABC/3TC/DTG	529	597	N
216	M	58	N		Y	42	88	Y	4	4	20	FTC/TAF/BIC	847	512	HyT
375	F	58	N		Y	42	47	Y	7	7	50	ABC/3TC/DTG	372	713	N
382	F	59	N		Y	42	46	Y	35	22	<20	ABC/3TC, EFV	1548	760	HyT, OUD
384	F	63	N		Y	44	52	Y	18	12	20	FTC, RAL, AZV	892	708	N
349	F	58	N		Y	35	50	Y	18	18	<20	FTC/TAF/BIC	503	755	D
584	F	57	N		Y	48	28	Y	16	16	20	FTC/TAF/COB /DRV	923	1169	N
303	F	36	N		Y	42	24	Y	5	5	<20	FTC/TAF/COB /DRV	570	585	HepB

ID	Sex	Age	COVID	Severity	Vaccination	Days between doses	Days post Boost/ Infection	HIV-Infection	Years infected	Years under therapy	Viral load	Therapy	CD4+ T-cells	CD8+ T-cells	Comorbidities
298	F	62	N		Y	45	38	Y	2	2	20	FTC/TDF, RAL	701	298	D
404	F	55	N		Y	28	30	Y	20	20	60	ABC/3TC/DTG	1182	719	A, LC, P
3	M	61	Y	2	N		23	N				None	N/A	N/A	N
12	F	65	Y	2	N		28	N				None	N/A	N/A	N
25	M	67	Y	2	N		28	N				None	N/A	N/A	N
32	M	66	Y	4	N		13	N				None	N/A	N/A	N
33	F	48	Y	4	N		7	N				None	N/A	N/A	N
38	F	50	Y	3	N		16	N				None	N/A	N/A	N
17	F	40	Y	2	N		20	N				None	N/A	N/A	N
255	M	56	Y	3	N		53	N				None	N/A	N/A	N
585	M	35	Y	2	N		240	N				None	N/A	N/A	N
140	M	55	Y	3	N		31	Y	6	5	<20	FTC/TAF/BIC	1034	643	N
62	M	59	Y	1	N		116	Y	12	12	<20	FTC/TAF/BIC	526	321	N
221	M	57	Y	1	N		63	Y	9	9	<20	FTC/TAF/BIC	1186	616	N
229	F	39	Y	3	N		46	Y	15	12	<20	FTC/TAF/RPV	859	586	ET
257	F	43	Y	3	N		29	Y	29	9	20	3TC/DTG	743	963	N
109	F	31	Y	2	N		27	Y	3	3	<20	DTG, FTC/TDF	605	490	N

ID	Sex	Age	COVID	Severity	Vaccination	Days between doses	Days post Boost/ Infection	HIV-Infection	Years infected	Years under therapy	Viral load	Therapy	CD4+ T-cells	CD8+ T-cells	Comorbidities
261	M	43	Y	3	N		77	Y	8	8	<20	FTC/TDF/RPV	940	685	N
510	M	43	Y	2	N		187	Y	17	6	20	FTC/TDF, DTG	708	578	N
51	M	57	Y	3	N		42	Y	6	6	<20	FTC/TAF/COB /EVG	322	936	N
231	F	38	Y	2	N		34	Y	13	1	<20	DRV, RTV, FTC/TDF	738	643	N
148	M	62	Y	3	N		28	Y	15	15	<20	FTC/TAF/COB /EVG	415	741	CHD, CKD, N, LF, PAOD
586	M	41	Y	1	N		264	Y	6	6	20	FTC/TAF/BIC	393	1023	N
406	F	63	Y	3	N		95	Y	11	8	<20	FTC/TAF/BIC	1324	395	N

[1]No Comorbidities stated for all healthy controls.

Abbreviations: M: male, F: female, N: No, Y: Yes, N/A: None available, Severity: 1 = Asymptomatic, 2 = Mild, 3 = Moderate, 4 = Severe (Hospitalization), A: Alcoholism, AA: Allergic asthma, ACL: Adenocarcinoma of the lung, AF: Atrial fibrillation, ASCT: Autologous stem cell transplantation, BL: B-cell lymphocytosis, CC: Chondrocalcinosis, CDK: Chronic kidney disease, CHD: Coronary heart disease, COPD: Chronic obstructive pulmonary disease, CT: Cyclothymia, D: Depression, DM1: Diabetes mellitus type 1, DM2: Diabetes mellitus type 2, ET: Essential thrombocythemia (untreated), HbH: Hemoglobin H disease with hemolytic anemia, HepB: Hepatitis B (treated), HPT: Hyperparathyroidism, HyT: Hypothyroidism, LC: Liver cirrhosis, LCpH: Liver cirrhosis post Hepatitis C, LF: Liver fibrosis, OUD: Opioid use disorder, P: Psoriasis, PAOD: Peripheral artery occlusive disease, PCBCL: Primary cerebral B-cell lymphoma, PF: Pulmonary fibrosis, SA: sleep apnea.
