# Supplementary material for: Characterization of Serum and Mucosal SARS-CoV-2-Antibodies in HIV-1-Infected Subjects after BNT162b2 mRNA Vaccination or SARS-CoV-2 Infection
Source: Viruses. 2022 Mar 21;14(3):651. doi: 10.3390/v14030651 (PMC8952283; doi:10.3390/v14030651)
Supplement: Supplementary file 1 [file viruses-14-00651-s001.zip › Suppl. Table S2.pdf]

Supplementary Table S2. Serum anti-spike IgA and IgG levels.

	IgG	IgA
**SARS-CoV-2 NI (n=6)**	0.2 (0.1-0.4)[1]	0.4 (0.1-0.8)
**Vaccinated HU (n=61)**	8.6 (7.7-9.5)	7.4 (4.5-8.4)
**Vaccinated HIV (n=50)**	8.0 (7.2-8.8)	5.8 (2.9-8.3)
**COVID HU (n=17)**	3.9 (2.6-6.0)	4.0 (2.5-7.6)
**COVID HIV (n=26)**	3.9 (2.2-6.6)	3.2 (1.5-6.7)
**P value vaccinated vs. COVID**		
**HU**	<0.0001	0.023
**HIV**	<0.0001	0.086
**P value HU vs. HIV**		
**Vaccinated**	0.028	0.077
**COVID**	0.816	0.522

[1] Shown are medians with IQRs in brackets of serum anti-spike IgG and IgA ratios. NI: non-immune, HU: HIV-1-uninfected. HIV: HIV-1-infected. A ratio of <0.8 was considered as negative, a ratio of 0.8-1.1 as borderline and ≥1.1 as positive. Significance was tested by Mann-Whitney-U test. All vaccinated and convalescent groups had significantly higher IgG- (p≤0.0001) and IgA-ratios (p≤0.0009) compared to the NI group.
