# Supplementary material for: Characterization of Serum and Mucosal SARS-CoV-2-Antibodies in HIV-1-Infected Subjects after BNT162b2 mRNA Vaccination or SARS-CoV-2 Infection
Source: Viruses. 2022 Mar 21;14(3):651. doi: 10.3390/v14030651 (PMC8952283; doi:10.3390/v14030651)
Supplement: Supplementary file 1 [file viruses-14-00651-s001.zip › Suppl. Table S3.pdf]

Supplementary Table S3. Neutralizing activity (NA) in serum at 1:5 and 1:101 dilution.

	n 1:5	NA 1:5	n 1:101	NA 1:101
**SARS-CoV-2 NI**	6	0.0% (0.0-9.6%)[1]	6	/
**Vaccinated HU**	57	99.0% (97.6-99.4%)	60	30.0% (4.1-62.3%)
**Vaccinated HIV**	50	98.7% (92.2-99.4 %)	50	11.9% (0.0-43.4%)
**COVID HU**	16	48.7% (17.0-71.3%)	17	/
**COVID HIV**	24	46.9% (15.8-83.1%)	26	/
**P value vaccinated vs. COVID**				
**HU**		<0.0001		/
**HIV**		<0.0001		/
**P value HU vs. HIV**				
**Vaccinated**		0.416		0.031
**COVID**		0.733		/

[1] Shown are medians with IQRs in brackets of neutralizing activity in serum. NI: non-immune, HU: HIV-1-uninfected. An inhibition of <20% was considered negative, an inhibition of 20-35% as borderline and ≥35% as positive. All vaccinated and convalescent groups showed significantly higher neutralizing activity compared to the NI group (p≤0.001).
