# Supplementary material for: Characterization of Serum and Mucosal SARS-CoV-2-Antibodies in HIV-1-Infected Subjects after BNT162b2 mRNA Vaccination or SARS-CoV-2 Infection
Source: Viruses. 2022 Mar 21;14(3):651. doi: 10.3390/v14030651 (PMC8952283; doi:10.3390/v14030651)
Supplement: Supplementary file 1 [file viruses-14-00651-s001.zip › Suppl. Table S4.pdf]

Suppl. Table S4: Multivariate linear regression analysis of serum spike-specific neutralizing antibody levels of SARS-CoV-2 vaccinated subjects compared to COVID-19 convalescent subjects

	Vaccinated HU		Vaccinated HIV	
**Variable**	**Estimates (95% CI)**	**p value**	**Estimates (95% CI)**	**p value**
**(Intercept)**	94.05 (87.86 to 100.23)	<0.0001	94.06 (87.89 to 100.23)	<0.0001
**Age, per decade**	-2.50 (-5.16 to 0.16)	0.065	-2.50 (-5.16 to 0.16)	0.065
**COVID HU**	-44.93 (-56.11 to -33.75)	<0.0001	-44.94 (-56.34 to -33.54)	<0.0001
**COVID HIV**	-37.67 (-47.88 to -27.45)	<0.0001	44.94 (-56.34 to -33.54)	<0.0001
**Female gender**	2.02 (-4.79 to 8.82)	0.559	2.02 (-4.79 to 8.82)	0.559
**Time after vaccine, per week**	-0.87 (-1.42 to -0.33)	0.002	-0.87 (-1.42 to -0.33)	0.002
