# Supplementary material for: Characterization of Serum and Mucosal SARS-CoV-2-Antibodies in HIV-1-Infected Subjects after BNT162b2 mRNA Vaccination or SARS-CoV-2 Infection
Source: Viruses. 2022 Mar 21;14(3):651. doi: 10.3390/v14030651 (PMC8952283; doi:10.3390/v14030651)
Supplement: Supplementary file 1 [file viruses-14-00651-s001.zip › Suppl. Table S5.pdf]

Supplementary Table S5. Saliva anti-spike IgA and IgG levels.

	IgA	Normalized IgA	IgG	Normalized IgG
**SARS-CoV-2 NI (n = 6)**	0.4 (0.1-0.6) [1]	0.1 (0.0-0.1)	0.1 (0.1-0.1)	0.1 (0.0-0.1)
**Vaccinated HU (n = 15)**	0.6 (0.4-1.9)	0.9 (0.3-1.3)	0.5 (0.4-1.3)	0.4 (0.2-1.3)
**Vaccinated HIV (n = 19)**	0.5 (0.3-1.3)	0.3 (0.2-0.8)	0.4 (0.2-0.6)	0.3 (0.2-0.4)
**COVID HU (n = 8)**	1.2 (0.5-1.7)	0.9 (0.5-1.4)	0.2 (0.1-0.4)	0.1 (0.1-0.4)
**COVID HIV (n = 13)**	1.6 (0.8-2.9)	0.8 (0.3-1.2)	0.2 (0.1-0.5)	0.1 (0.0-0.2)
**P value HU vs. HIV**				
**Vaccinated**	0.554	0.073	0.225	0.036
**COVID**	0.310	0.500	0.790	0.310
**P value Vaccinated vs. COVID**				
**HU**	0.789	0.548	0.020	0.024
**HIV**	0.059	0.211	0.070	0.001

[1] Shown are medians with IQRs in brackets of spike-specific IgA and IgG levels given as antibody ratios obtained by dividing the extinction of the sample by that of the calibrator. NI: non-immune, HU: HIV-1-uninfected. A ratio of <0.8 was considered as negative, a ratio of 0.8-1.1 as borderline and ≥1.1 as positive. Significance was tested by Mann-Whitney-U tests.
