# Supplementary material for: Characterization of Serum and Mucosal SARS-CoV-2-Antibodies in HIV-1-Infected Subjects after BNT162b2 mRNA Vaccination or SARS-CoV-2 Infection
Source: Viruses. 2022 Mar 21;14(3):651. doi: 10.3390/v14030651 (PMC8952283; doi:10.3390/v14030651)
Supplement: Supplementary file 1 [file viruses-14-00651-s001.zip › Suppl. Table S6.pdf]

Supplementary Table S6: Raw ELISA data

ID	Sample	IgG Ratio	IgA Ratio	% Inhibition
224	Serum	0.2	0.2	0.0
	Saliva	0.1	0.6	5.6
539	Serum	0.3	0.4	10.8
	Saliva	0.1	0.1	21.0
93	Serum	0.6	0.4	0.0
	Saliva	0.1	0.6	0.0
540	Serum	0.1	1.9	0.0
	Saliva	0.1	0.1	2.2
356	Serum	0.1	0.1	9.6
	Saliva	0.1	0.3	2.3
70	Serum	0.1	0.4	0.0
	Saliva	0.1	0.6	16.7
254	Serum	8.4	4.0	99.4
	Saliva	1.0	1.0	15.6
264	Serum	9.7	12.2	99.4
	Saliva	4.0	4.3	24.6
269	Serum	9.9	7.5	99.1
	Saliva	1.3	1.6	8.0
265	Serum	9.1	7.4	98.5
	Saliva	0.2	0.5	8.7
268	Serum	9.5	7.4	99.3
	Saliva	1.6	3.9	20.2
266	Serum	9.5	7.0	99.5
	Saliva	1.1	1.9	0.0
267	Serum	10.6	7.3	99.7
	Saliva	0.4	0.4	0.0
271	Serum	10.2	7.4	99.8
	Saliva	5.3	5.4	10.0
270	Serum	8.2	1.6	94.0
	Saliva	0.0	0.4	0.0
6	Serum	9.2	7.4	99.0
	Saliva	0.4	0.3	5.8

272	Serum	9.8	4.4	98.4
	Saliva	0.4	0.6	24.7
8	Serum	9.4	5.8	99.0
	Saliva	0.7	0.5	5.4
5	Serum	8.5	4.7	97.8
	Saliva	0.2	0.3	1.9
15	Serum	8.1	6.1	93.6
	Saliva	0.5	0.4	5.0
22	Serum	9.7	3.2	90.0
	Saliva	0.4	0.6	10.2
526	Serum	7.9	1.8	97.2
	Saliva	2.3	1.3	0.0
56	Serum	6.9	1.7	94.0
	Saliva	0.4	0.4	0.0
74	Serum	8.3	1.3	96.9
	Saliva	0.2	0.3	0.0
232	Serum	10.9	4.5	99.8
	Saliva	5.6	7.8	4.7
383	Serum	8.7	3.0	98.6
	Saliva	0.6	0.5	0.0
275	Serum	7.5	6.2	92.5
	Saliva	0.3	0.7	16.2
420	Serum	5.1	8.4	63.8
	Saliva	0.1	0.6	38.5
45	Serum	7.6	11.9	99.3
	Saliva	0.5	0.5	0.0
496	Serum	7.2	2.2	90.0
	Saliva	0.3	0.8	0.0
240	Serum	9.1	11.1	99.4
	Saliva	0.5	3.3	6.5
503	Serum	7.4	2.7	87.1
	Saliva	0.2	0.2	21.5
98	Serum	7.5	7.0	99.1
	Saliva	0.1	0.3	25.4
390	Serum	5.9	1.5	88.6

	Saliva	0.6	1.4	3.3
289	Serum	10.3	8.1	98.4
	Saliva	0.2	0.3	1.0
436	Serum	8.0	2.5	94.6
	Saliva	0.8	0.6	0.0
396	Serum	7.2	2.5	95.9
	Saliva	0.8	0.4	14.7
218	Serum	7.5	5.0	99.0
	Saliva	0.2	0.5	17.9
297	Serum	8.5	3.5	97.9
	Saliva	0.4	1.6	15.8
441	Serum	4.6	2.7	69.4
	Saliva	0.2	0.4	7.4
256	Serum	3.0	2.6	64.6
	Saliva	0.3	2.6	0.0
263	Serum	2.6	3.5	58.2
	Saliva	1.0	1.8	0.0
565	Serum	5.4	4.0	78.8
	Saliva	0.1	1.0	3.2
566	Serum	2.7	3.3	39.8
	Saliva	0.1	1.3	23.3
567	Serum	5.5	2.5	34.0
	Saliva	0.1	1.1	30.9
307	Serum	3.5	1.9	16.8
	Saliva	0.1	0.3	14.0
568	Serum	9.1	9.7	96.7
	Saliva	0.4	1.2	0.0
569	Serum	3.9	1.7	51.9
	Saliva	0.2	0.2	0.0
113	Serum	1.3	0.3	0.0
	Saliva	0.1	0.2	0.8
334	Serum	2.5	7.9	47.7
	Saliva	0.1	1.1	7.3
532	Serum	1.3	0.4	14.7
	Saliva	0.1	0.5	5.8

501	Serum	5.0	3.2	93.7
	Saliva	1.5	6.2	20.4
230	Serum	6.9	5.2	97.7
	Saliva	0.5	1.5	9.9
120	Serum	2.4	2.4	46.1
	Saliva	0.1	1.0	1.1
178	Serum	0.6	0.4	8.5
	Saliva	0.0	0.3	0.0
492	Serum	4.4	2.5	66.6
	Saliva	0.1	1.6	5.5
491	Serum	7.2	5.4	99.4
	Saliva	0.5	2.7	9.7
422	Serum	6.6	7.0	88.3
	Saliva	0.5	3.4	0.0
552	Serum	4.0	4.1	56.0
	Saliva	0.2	2.0	23.1
260	Serum	3.7	3.3	40.8
	Saliva	0.3	2.1	4.5
564	Serum	6.7	7.7	82.5
	Saliva	0.3	3.2	17.5
1	Serum	8.4	10.0	99.5
247	Serum	7.6	10.4	99.6
248	Serum	7.7	10.3	99.6
251	Serum	8.6	10.3	99.4
252	Serum	9.6	8.2	99.8
9	Serum	9.7	8.0	98.6
514	Serum	10.2	9.2	98.9
516	Serum	8.2	1.3	95.9
517	Serum	8.6	3.0	95.1
528	Serum	9.1	10.3	98.4
475	Serum	10.8	9.0	99.1
580	Serum	6.8	9.9	98.5

4083	Serum	9.5	8.5	99.6
4439	Serum	8.2	7.7	99.2
3012	Serum	8.8	4.4	99.1
3865	Serum	7.8	8.1	98.6
3815	Serum	6.3	7.1	84.5
4402	Serum	3.8	0.5	40.7
2918	Serum	9.1	8.8	99.4
4628	Serum	8.9	5.5	98.4
908	Serum	7.0	7.0	97.8
3827	Serum	7.1	3.9	87.1
89	Serum	3.0	3.8	34.9
770	Serum	10.5	8.3	99.6
773	Serum	12.6	5.2	99.3
781	Serum	10.3	3.4	99.0
9240	Serum	6.2	4.7	89.3
4432	Serum	9.6	8.4	98.3
1452	Serum	7.1	8.3	99.2
1674	Serum	8.1	5.0	98.3
2998	Serum	9.3	3.7	99.3
3959	Serum	8.8	8.3	99.4
2799	Serum	8.2	8.6	97.2
7673	Serum	8.8	8.3	99.6
2960	Serum	8.0	3.3	98.2
1694	Serum	7.5	8.2	98.4
3830	Serum	8.3	8.4	99.6
1657	Serum	5.2	1.6	88.1
2974	Serum	9.0	5.0	99.4
1827	Serum	7.8	7.0	N/A
7864	Serum	7.3	5.8	N/A
530	Serum	5.9	8.6	N/A

273	Serum	6.8	5.6	97.4
487	Serum	9.2	0.0	97.3
23	Serum	8.3	10.0	99.2
538	Serum	10.1	2.9	99.3
469	Serum	8.3	10.3	99.4
305	Serum	3.7	8.0	32.4
562	Serum	7.0	3.0	97.1
563	Serum	5.5	4.0	94.2
141	Serum	8.3	8.3	99.6
143	Serum	9.0	9.4	99.6
183	Serum	9.0	9.7	99.9
462	Serum	8.2	6.3	98.5
340	Serum	8.5	6.0	99.4
571	Serum	8.1	6.6	99.1
329	Serum	9.1	8.7	99.4
65	Serum	9.4	10.0	99.9
119	Serum	8.7	5.5	98.7
154	Serum	5.0	3.0	71.4
336	Serum	9.4	7.4	99.2
509	Serum	9.3	8.4	100.0
323	Serum	8.6	10.3	99.2
485	Serum	7.6	6.3	98.7
158	Serum	8.0	8.5	96.3
239	Serum	6.8	2.6	99.4
46	Serum	5.0	0.7	59.9
216	Serum	7.6	3.9	91.3
375	Serum	8.4	5.9	99.7
382	Serum	6.1	1.7	75.1
384	Serum	8.5	6.0	99.7
349	Serum	7.2	3.2	89.3

584	Serum	10.5	6.3	99.5
303	Serum	10.1	8.5	99.9
298	Serum	7.7	5.7	79.3
404	Serum	8.6	5.1	99.1
3	Serum	5.3	3.4	17.8
12	Serum	5.8	10.0	48.3
25	Serum	2.6	4.6	0.0
32	Serum	6.2	7.8	49.2
33	Serum	0.3	4.6	0.0
38	Serum	7.9	7.3	73.5
17	Serum	2.1	8.3	12.8
255	Serum	6.9	5.5	94.4
585	Serum	0.4	1.0	N/A
140	Serum	1.3	5.6	27.9
62	Serum	1.5	1.3	0.0
221	Serum	3.2	6.2	16.3
229	Serum	5.2	6.4	31.9
257	Serum	10.2	10.1	97.9
109	Serum	4.9	9.9	79.9
261	Serum	3.0	1.5	15.6
510	Serum	1.4	1.4	0.0
51	Serum	4.1	2.80	45.6
231	Serum	3.4	N/A	N/A
148	Serum	8.3	10.2	5.2
586	Serum	3.1	1.3	0.0
406	Serum	6.6	3.1	0.0

Abbreviations: N/A: None available.
