# Supplementary figures and images for: Characterization of Serum and Mucosal SARS-CoV-2-Antibodies in HIV-1-Infected Subjects after BNT162b2 mRNA Vaccination or SARS-CoV-2 Infection
Source: Viruses. 2022 Mar 21;14(3):651. doi: 10.3390/v14030651 (PMC8952283; doi:10.3390/v14030651)

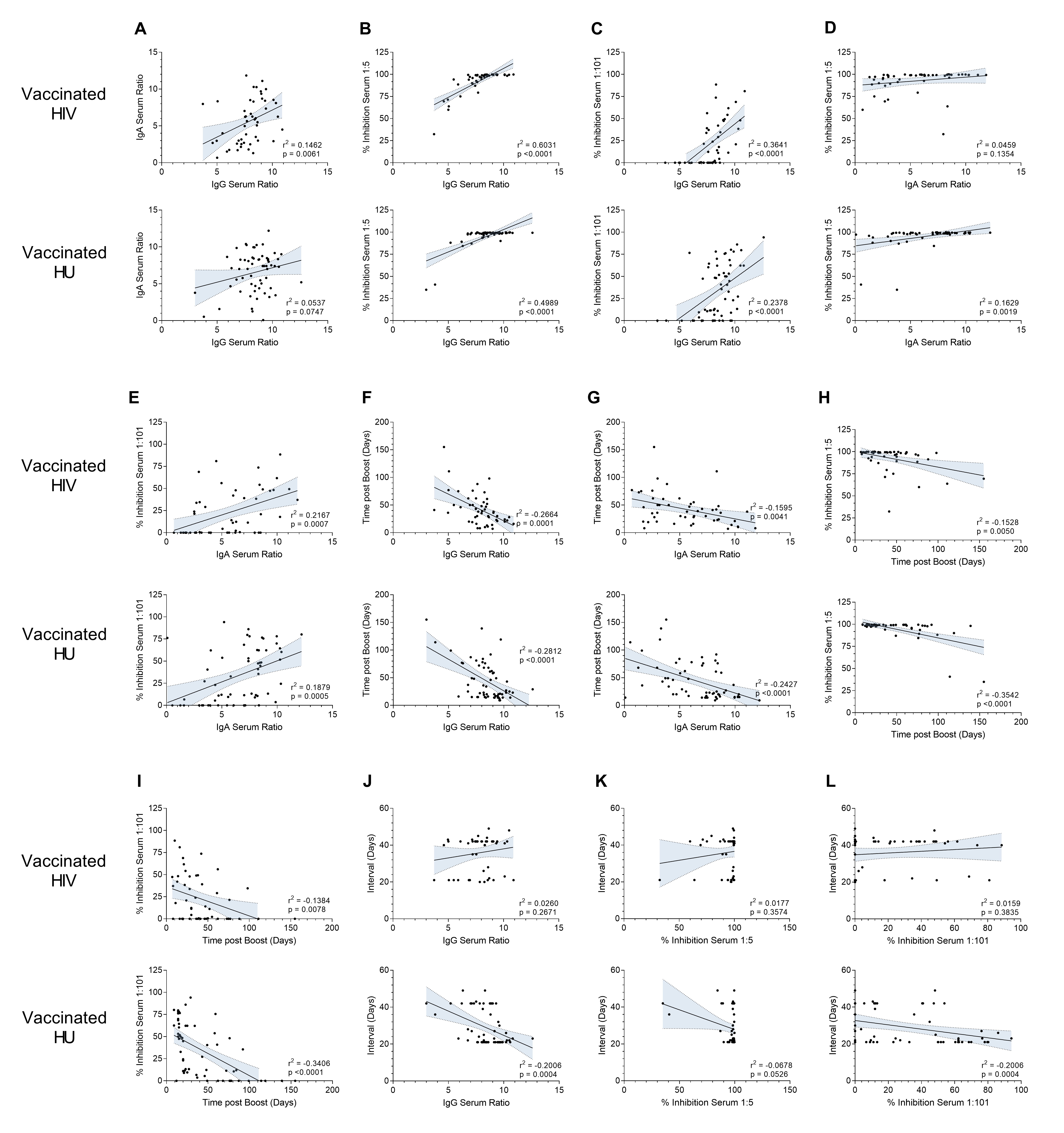

Supplement: Supplementary file 1 [file viruses-14-00651-s001.zip › Suppl. Figure S1 .tif]

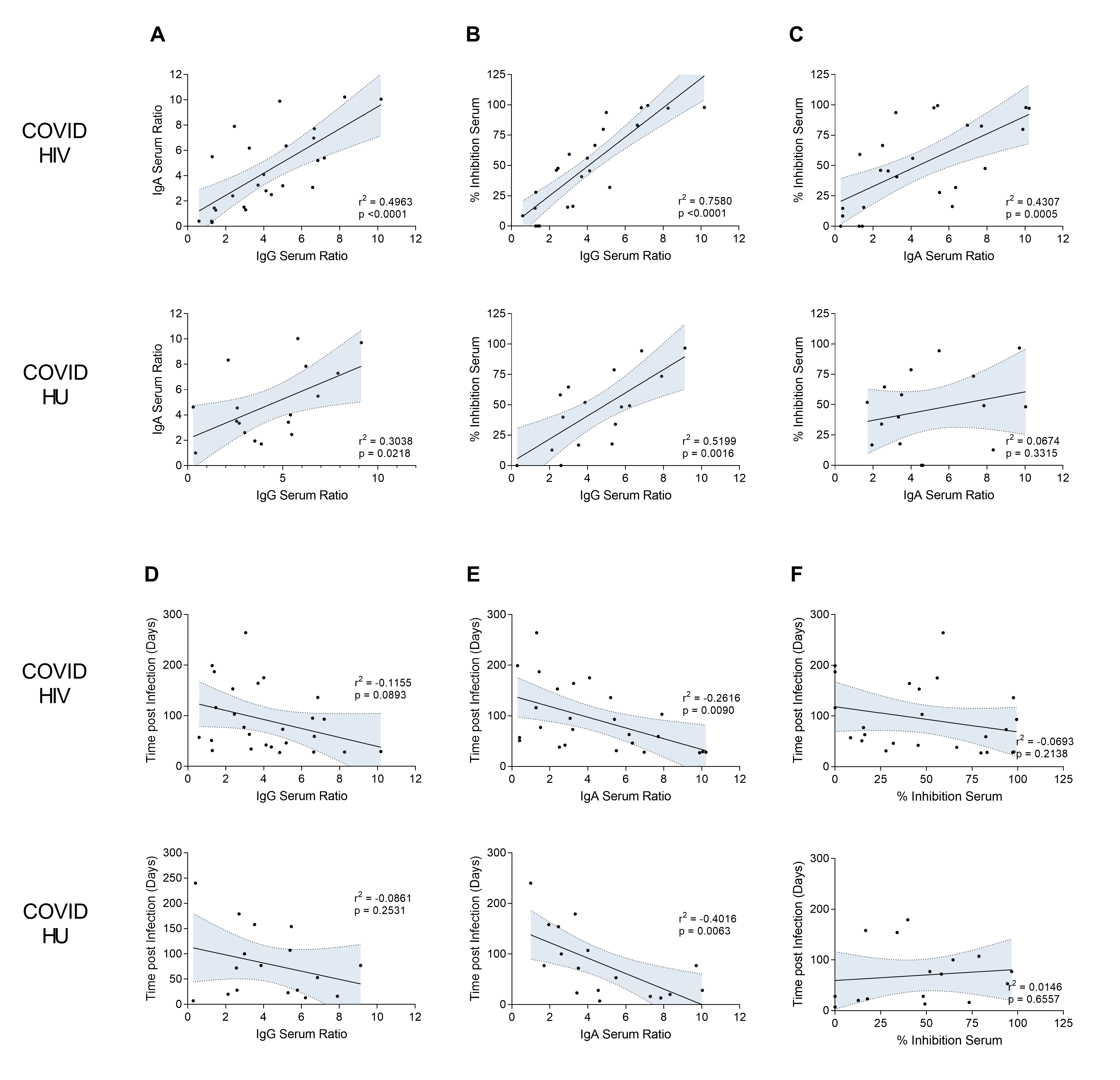

Supplement: Supplementary file 1 [file viruses-14-00651-s001.zip › Suppl. Figure S2 .tif]
